# Prevalence and dynamics of clonal hematopoiesis caused by leukemia-associated mutations in elderly individuals without hematologic disorders

**DOI:** 10.1038/s41375-020-0869-y

**Published:** 2020-05-26

**Authors:** Danica Midic, Jenny Rinke, Florian Perner, Violetta Müller, Anna Hinze, Frank Pester, Jürgen Landschulze, Jana Ernst, Bernd Gruhn, Georg Matziolis, Florian H. Heidel, Andreas Hochhaus, Thomas Ernst

**Affiliations:** 1 Abteilung Hämatologie und Internistische Onkologie, Klinik für Innere Medizin II, Universitätsklinikum Jena, Jena, Germany; 2 HausArztZentrum, Kahla, Germany; 3 Klinik für Kinder- und Jugendmedizin, Universitätsklinikum Jena, Jena, Germany; 4 Orthopädische Klinik der Waldkliniken Eisenberg, Eisenberg, Germany; 5 Leibniz Institute on Aging, Fritz-Lipmann-Institute, Jena, Germany

**Keywords:** Leukaemia, Cancer genetics

## Abstract

Clonal hematopoiesis is frequently observed in elderly people. To investigate the prevalence and dynamics of genetic alterations among healthy elderly individuals, a cohort of 50 people >80 years was genotyped for commonly mutated leukemia-associated genes by targeted deep next-generation sequencing. A total of 16 somatic mutations were identified in 13/50 (26%) individuals. Mutations occurred at low variant allele frequencies (median 11.7%) and remained virtually stable over 3 years without development of hematologic malignancies in affected individuals. With *DNMT3A* mutations most frequently detected, another cohort of 160 healthy people spanning all age groups was sequenced specifically for *DNMT3A* revealing an overall mutation rate of 6.2% (13/210) and an age-dependent increase of mutation prevalence. A significant difference (*p* = 0.017) in the *DNMT3A* expression pattern was detected between younger and healthy elderly people as determined by qRT-PCR. To evaluate the selection of clonal hematopoietic stem cells (HSCs), bone marrow of two healthy individuals with mutant *DNMT3A* was transplanted in a humanized mouse model. Xenografts displayed stable kinetics of *DNMT3A* mutations over 8 months. These findings indicate that the appearance of low-level clones with leukemia-associated mutations is a common age-associated phenomenon, but insufficient to initiate clonal selection and expansion without the additional influence of other factors.

## Introduction

Clonal hematopoiesis (CH) is defined as the clonal expansion of blood cells derived from a single hematopoietic stem cell. Recent studies suggest that CH is an age-related event, triggered by somatic mutations in leukemia-associated genes. While acquisition of these mutations is considered one of the earliest events in the pathogenesis of different hematologic malignancies, clonal hematopoiesis driver mutations (CHDMs) may be present and cause CH without any clinically distinct phenotype. Three recent whole-exome-sequencing analyses of large populations have revealed age-related hematopoietic clones in apparently healthy individuals, driven by mutations of genes recurrently mutated in myeloid neoplasms and associated with increased risk of hematologic cancer and cardiovascular diseases [1–3]. CH with somatic mutations was observed in 10% of individuals older than 65 years of age, but only in 1% of those younger than 50 years of age [2]. Furthermore, CHDMs were detected in 10% of individuals older than 70 years and in about 20% of those older than 90 years [3]. The most frequently mutated genes include *DNMT3A*, *TET2*, *JAK2*, and *ASXL1*. Based on these three large studies, Steensma et al. proposed the term CHIP (clonal hematopoiesis of indeterminate potential) for individuals harboring somatic mutations of genes frequently mutated in hematologic malignancies with a variant allele frequency (VAF) ≥ 2%, in the absence of definitive morphologic and clinical evidence of a hematologic malignancy [4].

The fact that patients with hematologic malignancies and healthy individuals share a common set of genetic mutations highlights the need to further elucidate the prevalence and kinetics of these mutations within the general population and to understand the underlying mechanisms of clonal selection and stability. This study investigated the prevalence and long-term dynamics of genetic alterations in leukemia-associated genes among elderly individuals without hematologic disorders or cancer. As *DNMT3A* mutations were most frequently detected, blood samples of 160 individuals of all ages of the general population were analyzed for *DNMT3A* mutations. Additionally, relative *DNMT3A* expression levels of younger and older individuals were compared to investigate age-related differences beyond the mutation status. A model of *DNMT3A* activity in hematopoietic stem cells (HSCs) proposed by Challen et al. suggests the crucial role of *DNMT3A* in balancing differentiation and self-renewal of HSCs by upregulation of HSC multipotency genes and downregulation of differentiation genes. Lack of *DNMT3A* function was shown to lead to altered methylation patterns reminiscent of those observed in human malignancies and an increased expression of genes normally restricted to stem cells [5]. Considering the key epigenetic regulatory role and the frequent involvement of DNMT3A in human malignancies, the study presented here finally investigated clonal expansion using bone marrow of healthy individuals with mutant *DNMT3A* in a humanized mouse model.

## Materials and methods

### Cohorts of healthy individuals

The first cohort included 50 elderly individuals (male, *n* = 21; median age 84 years, range 80–90 years). Peripheral blood (PB) samples were taken and sequenced for a panel of commonly mutated leukemia-associated genes. The second cohort of 160 healthy people <80 years of age was specifically sequenced for *DNMT3A* mutations (male, *n* = 77; median age 40 years, range 0–79 years). Total leukocytes were isolated from PB after red cell lysis. As a source of constitutional DNA, oral mucosa cells were obtained from all patients using buccal swabs. All procedures were in accordance with the ethical standards of the institutional research committee and with the Declaration of Helsinki. Informed consent was obtained from all individuals included in the study.

### DNA and RNA extractions

Genomic DNA (gDNA) was isolated from PB leukocytes and oral mucosa cells using the QIAamp DNA Mini Kit (Qiagen, Hilden, Germany) according to the manufacturer’s recommendations. For RNA isolation, 1 ml Trizol^®^ reagent (Thermo Fisher Scientific, Waltham, MA, USA) was added to 2 × 10^7^ cells and mixed thoroughly. Subsequent RNA extraction was performed as described in the literature [6].

### Next-generation sequencing (NGS)

Samples of 50 healthy elderly individuals were genotyped for a panel of 30 commonly mutated leukemia-associated genes by targeted deep NGS [7, 8], using the 454 GS Junior platform (Roche Diagnostics, Mannheim, Germany) at a sensitivity level of 5%. Prior to sequencing, whole genome amplification (WGA) was performed using 20 ng template gDNA and the Repli-g Ultra Fast Mini Kit (Qiagen) in accordance with manufacturer's instructions. Analyses covered genes involved in signaling, transcription, epigenetics, RNA splicing, and the cohesin complex. Another cohort of 160 healthy people <80 years of age was specifically sequenced for *DNMT3A* mutations, using a custom-designed highly sensitive *DNMT3A*-specific deep NGS assay (sensitivity 1%) covering the entire coding region of the *DNMT3A* gene (exons 2–23). For each NGS run 8 *JAK2* p.V617F samples were generated and used as an external sensitivity control. PCR products were resolved on 3% agarose gels and visualized by staining with ethidium bromide. Mutations found in WGA samples were verified using gDNA isolated from leukocytes and buccal swabs to confirm the somatic origin of mutations, in a separate NGS run. All mutational percentages listed refer to the results found in gDNA. NGS data was analyzed with the GS Amplicon Variant Analyzer (AVA) software (version 2.7; Roche). The open access tools Protein Variation Effect Analyzer (PROVEAN) and Polymorphism Phenotyping v2 (PolyPhen-2) were used for evaluation of potential consequences of the identified mutations [9, 10].

### Expression analysis by quantitative real-time PCR (qRT-PCR)

For *DNMT3A* gene expression analyses, cDNA synthesis was performed using M-MLV Reverse transcriptase (Invitrogen GmbH, Karlsruhe, Germany) and a minimum of 1 µg RNA. Analysis by qRT-PCR was performed according to standard protocols using SYBR Green I (Roche), *DNMT3A* as a target, and *β-glucuronidase* (*GUSB*) as a reference gene. The relative expression rate was calculated using the ΔCt-method [11]. Primers were designed using primer3web (version 4.0.0). Primer sequences with the corresponding product size are shown in Table SI. Statistical analysis was performed using the Mann–Whitney nonparametric test, provided by GraphPad Prism v6.01 (GraphPad Software, Inc., San Diego, CA, USA).

### Patient-derived xenograft (PDX) model

Bone marrow cells of two healthy elderly individuals (age 69 and 79 years) with *DNMT3A* mutations were transplanted into a humanized mouse model. Primary bone marrow cells of elderly healthy donors were obtained from fractured hip bones after hemiarthroplasty following informed consent according to the Helsinki declaration (approved by the local ethics committee #4753/04-16). Bone marrow cells were extracted from the bone, erythrolysed and cryopreserved in 1x freezing medium (80% FBS + 10% DMSO + 10% IMDM medium). NOD.Cg-Prkdc^scid^ Il2rg^tm1Wjl^ Tg(CMV-IL3, CSF2, KITLG) 1Eav/MloySzJ (NSGS) were obtained from The Jackson Laboratory (Bar Harbor, ME, USA). Before transplantation, 8–12-week-old adult mice were irradiated with 2 Gy (single dose). Via the tail vein, 2 × 10^6^ cells were injected intravenously. Engraftment of human cells (hCD45+) was analyzed by flow cytometry. Three mice were engrafted for each mutation. The *DNMT3A* mutations were previously detected using the MiniSeq System (TruSight Myeloid Sequencing Panel, VariantStudio Software 3.0, Illumina, San Diego, USA). For analysis, murine bone marrow cells were isolated from the femurs as previously described [12]. Bone marrow samples were then assessed for clonal selection of the known *DNMT3A* mutations by pyrosequencing, according to standard protocols. Sequencing primers are listed in the Table SII.

## Results

### Somatic mutations in healthy elderly individuals

A total of 16 somatic mutations in leukemia-associated genes were identified in 13 of 50 (26%) hematologically normal elderly individuals (Table 1). One subject presented with two and another subject with three mutations of different genes. Ten of 16 mutations (62.5%) affected epigenetic modifier genes (*DNMT3A*, *n* = 8; *TET2*, *n* = 1; *IDH2*, *n* = 1), four somatic mutations affected genes involved in the RNA splicing machinery (*SRSF2*, *n* = 2; *SF3B1*, *n* = 1; *U2AF1*, *n* = 1). Mutations in *TP53* and *NRAS* were identified in two individuals. Detected mutations were verified on non-amplified gDNA by performing a separate NGS run, whilst the somatic origin was confirmed using corresponding buccal swab gDNA. All but one mutation were missense mutations with cytosine to thymine transitions being the most common base pair change (*n* = 7). Mutations occurred at low VAF with a median of 11.7% (range, 1.0–30.7%), indicating that mutations were present in only a subset of blood cells.

**Table 1:** Somatic mutations in leukemia-associated genes within a cohort of 50 healthy elderly individuals.

Sample number	Sex, age	Gene	Variant Ensemble	Protein	% Reads displaying variant gDNA	% Reads displaying variant buccal cells	% Reads displaying variant gDNA (2-year follow-up)	% Reads displaying variant gDNA (3-year follow-up)
#5	Female, 86	*DNMT3A*	c.1403A>G	p.K468R	5.53	0.00	5.42	4.85
#9	Male, 81	*SRSF2*	c.144_169del(26)	p.Y50LfsX11	10.63	0.46	7.12	8.33
#10	Male, 86	*SF3B1*	c.2098A>G	p.K700E	6.56	0.00	†	†
#15	Female, 83	*DNMT3A*	c.2644C>T	p.R882C	4.89	0.00	6.76	─
		*DNMT3A*	c.2711C>T	p.P904L	3.60	0.00	3.54	─
#21	Male, 85	*IDH2*	c.418C>T	p.R140W	22.75	12.47	28.98	33.69
		*SRSF2*	c.284C>T	p.P95L	28.78	15.6	35.29	56.59
		*TET2*	c.5618T>C	p.I1873T	20.41	12.74	21.80	21.51
#22	Female, 85	*DNMT3A*	c.1988C>T	p.S663W	1.04	0.00	1.02	0.86
#24	Male, 81	*TP53*	c.638G>A	p.R213Q	15.43	0.90	15.70	18.50
#25	Male, 81	*DNMT3A*	c.2401A>G	p.M801V	30.69	10.82	30.04	32.15
#35	Female, 86	*NRAS*	c.35G>A	p.G12D	6.19	0.39	11.72	─
#37	Female, 81	*DNMT3A*	c.1031T>C	p.L344P	5.03	1.58	5.23	5.17
#41	Female, 82	*DNMT3A*	c.929T>C	p.I310T	17.82	0.00	17.26	21.60
#42	Male, 85	*U2AF1*	c.101C>T	p.S34F	23.16	1.38	†	†
#43	Female, 81	*DNMT3A*	c.1988C>T	p.S663W	12.67	0.00	14.38	18.80

† Patient deceased.

### Mutation dynamics and clinical outcome during a 3-year period

**Fig. 1:**
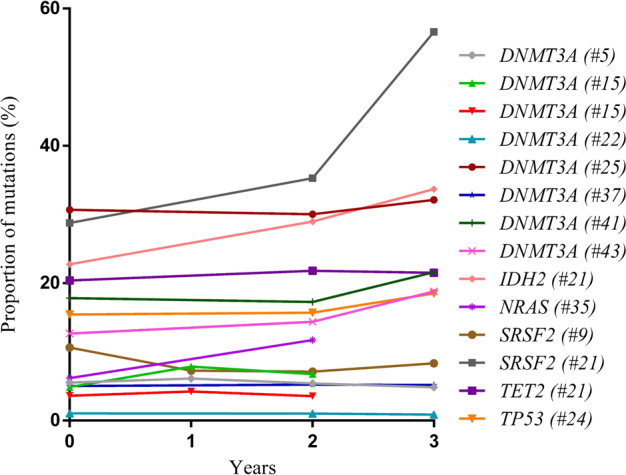
Dynamics of somatic mutations detected in 50 healthy elderly individuals during a 3-year follow up period. Two subjects with mutations in splicing factor genes *SF3B1* and *U2AF1* died. For two patients (#15 and #35) the 3-year follow-up samples were not available.

Mutation kinetics remained virtually stable over 3 years, with a median VAF of 13.1% (range, 1.0–35.3%) after a 2-year follow-up and a median VAF of 18.8% after a 3-year follow-up (range, 0.9–56.6%) (Table 1). The mutations’ dynamics during the 3-year period are depicted in Fig. 1. During the 3-year follow-up observation, two subjects with mutations in splicing factor genes died. One subject with an *SF3B1* mutation developed pancreatic cancer, the other subject harboring a *U2AF1* mutation died due to a stroke. All other individuals with mutations were alive without any evidence for a hematologic or oncologic disorder.

### Age-associated increase of somatic *DNMT3A* mutations in healthy individuals

**Fig. 2:**
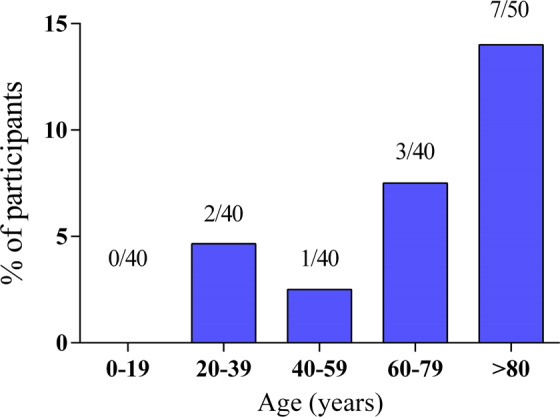
Age-associated increase of *DNMT3A* mutations in healthy individuals. Frequency of *DNMT3A* mutations was analyzed within five age cohorts (0–19; 20–39; 40–59; 60–79; >80 years) by a highly sensitive *DNMT3A*-specific deep next-generation sequencing assay.

Since *DNMT3A* mutations were the most common mutations identified in elderly individuals (8/16 detected mutations), we sought to study the age-dependent prevalence of *DNMT3A* mutations in more detail. Therefore, an additional cohort of 160 individuals was investigated, including healthy people <80 years of age (male, *n* = 77; median age 40 years, range 0–79 years) using a custom-designed highly sensitive *DNMT3A*-specific deep NGS assay (sensitivity 1%), covering the complete coding region of the *DNMT3A* gene (exons 2–23). The youngest individual with a *DNMT3A* mutation was a 28 years old female. Mutations occurred at low VAFs with a median of 8.2% (range, 1.5–37.3%). No *DNMT3A* mutation was detected in 40 analyzed PB samples of children and adolescents (0–19 years). Somatic *DNMT3A* mutations were found in 2/40 (5.0%) individuals with an age of 20–39 years, 1/40 subjects (2.5%) between 40 and 59 years, and in 3/40 subjects (7.5%) between 60 and 79 years of age (Fig. 2). Overall, six somatic *DNMT3A* mutations were identified in 6 of 160 (3.8%) individuals (Table 2) <80 years of age. In comparison, the mutation frequency of *DNMT3A* in healthy elderly persons (≥80 years) was with 14% (7/50) much higher. The *DNMT3A* mutation rate in the whole cohort of healthy individuals, both younger and elderly, was 6.2% (13/210) in total.

**Table 2:** Somatic *DNMT3A* mutations within a cohort of 160 healthy younger individuals.

Sample number	Sex, age	*DNMT3A* exon	Variant Ensemble	Protein	Ensemble entry	PolyPhen-2 prediction	PROVEAN prediction	% Reads displaying variant gDNA	% Reads displaying variant buccal cells
#72	Female, 28	23	c.2645G>A	p.R882H	Somatic mutation found in human cancers (COSM52944) and pathogenic SNP (rs147001633)	Probably damaging	Deleterious	18.88	6.10
#76	Male, 31	14	c.1651A>G	p.N551D	Not displayed in Ensemble	Probably damaging	Deleterious	8.49	0.00
#96	Male, 49	19	c.2309C>T	p.S770L	Somatic mutation found in human cancers (COSM231549) and SNP (rs758845779)	Probably damaging	Deleterious	2.43	0.00
#149	Male, 63	23	c.2644C>T	p.R882C	Somatic mutation found in human cancers (COSM53042) and pathogenic SNP (rs377577594)	Probably damaging	Deleterious	8.00	0.00
#151	Female, 69	8	c.862delC	p.R288Gfs*28	Not displayed in Ensemble	No predictions available	No predictions available	37.30	0.00
#158	Female, 74	20	c.2381T>C	p.F794S	Not displayed in Ensemble	Probably damaging	Deleterious	1.54	0.00

### *DNMT3A* mRNA expression in healthy individuals

**Fig. 3:**
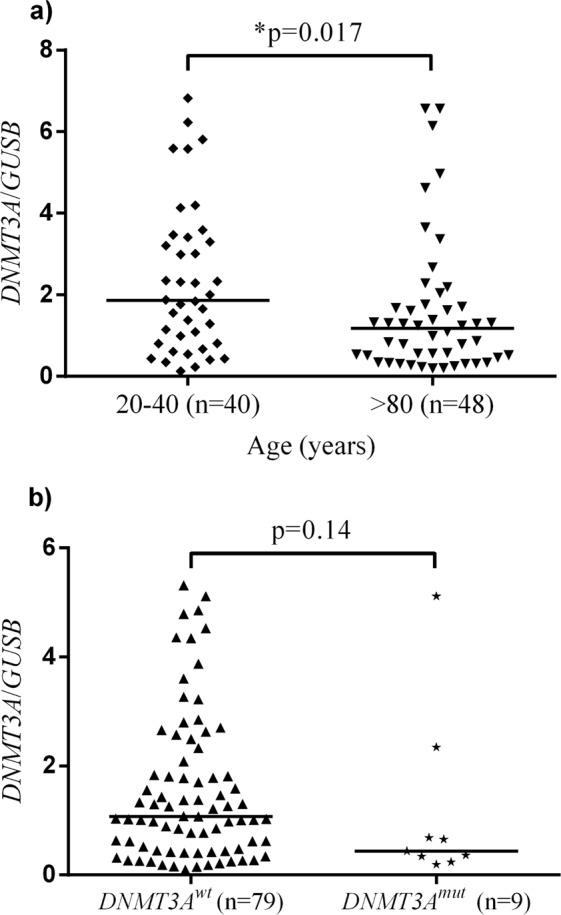
*DNMT3A* mRNA expression in healthy individuals. **a** Age-associated decrease of *DNMT3A* expression (median value depicted; *p = 0.017). **b** A marginal albeit not significant difference between *DNMT3A*^wt^ and *DNMT3A*^mut^ expression (median value depicted; *p* = 0.14). Statistical analysis was performed with the Mann–Whitney nonparametric test. wt wild type; mut mutant.

*DNMT3A* mRNA expression was analyzed by qRT-PCR in the previously sequenced cohort of younger healthy individuals (20–39 years, *n* = 40) and healthy elderly (≥80 years, *n* = 48). Significantly lower expression (*p = 0.017) was found in healthy elderly people (Fig. 3a). Taking sequencing results of these two groups into consideration, a marginal albeit not significant difference (*p* = 0.14) was found in expression levels comparing individuals carrying wild type *DNMT3A* (*DNMT3A*^*wt*^, *n* = 79) with those carrying *DNMT3A* somatic mutations (*DNMT3A*^*mut*^, *n* = 9) (Fig. 3b).

### Stable kinetics of *DNMT3A* mutations in a PDX model

To further investigate the clonal selection of *DNMT3A* mutations, transplantation of bone marrow cells of two healthy elderly individuals with different somatic *DNMT3A* mutations in a PDX model were performed. Mutations were detected using the MiniSeq System (Illumina), as a part of a subproject analyzing CHDM in bone marrow samples of healthy individuals. Detected *DNMT3A* mutations are shown in Table SIII. Mutations in human bone marrow cells were present at low VAFs of 23.1% and 6.3%, respectively (Table 3). Each human sample was transplanted into three mice. After 8 months, bone marrow samples were isolated from recipient mice, representing an aging mouse model of the hematopoietic compartment. The first *DNMT3A* mutation with VAF of 23.1% remained stable during this time in all three recipient mice, with a median VAF of 21.8% (range, 10.8–30.8%). The second *DNMT3A* mutation, initially present with a VAF of 6.3%, displayed stable mutation kinetic in one mouse, whereas in the other two mice no mutation was detectable (Table 3).

**Table 3:** *DNMT3A* mutation rate in bone marrow of healthy individuals vs. mice xenografts.

	Individual A	Mice xenografts (8 months after transplantation)		
		Mouse A1	Mouse A2	Mouse A3
*DNMT3A*^*mut*^ (S837X)				
VAF (%)	23.1	23.8	10.8	30.8

	Individual B	Mice xenografts (8 months after transplantation)		
		Mouse B1	Mouse B2	Mouse B3
*DNMT3A*^*mut*^ (D768H)				
VAF (%)	6.3	6.2	0	0

*VAF* variant allele frequency.

## Discussion

In this study, leukemia-associated mutations were frequently found in healthy elderly individuals. This confirms findings of recent exome-analysis studies who have shown age-related CH in healthy individuals, driven by mutations of genes recurrently mutated in myeloid neoplasms and associated with an increased risk of hematologic cancer and cardiovascular disease. All those studies identified similar genes, with the majority of mutations affecting epigenetic modifiers, such as *DNMT3A*, *TET2*, and *ASXL1* [1–3, 13]. A recent whole-genome sequencing approach on a large population cohort from Iceland also supports the finding that CH is very common in elderly, both with and without candidate driver mutations, suggesting that age-related CH is inevitable [14]. Here, mutations were detected predominantly in epigenetic modifier genes *DNMT3A*, *TET2*, and *IDH2*, which are known to promote self-renewal and block differentiation of HSCs. These CHDMs are considered to be founder events in the evolution of myeloid diseases, such as acute myeloid leukemia (AML), myelodysplastic syndrome (MDS), chronic myelomonocytic leukemia (CMML), and myeloproliferative neoplasms (MPN) [15, 16]. Defects in RNA splicing genes, such as *SF3B1*, *SRSF2*, and *U2AF1* have been described in pathogenesis of MDS [17], and were also detected within the cohort investigated.

In the study presented here, it was shown that mutation kinetics remain virtually stable over a 3 years follow-up. This suggests that detected CHDMs in healthy people enter a stage of clonal size stability for most people. A recent longitudinal study also showed temporal stability of mutated clones in healthy individuals [18], indicating an occurrence of these mutations in long-lived HSCs or committed progenitors. However, presence of these CHDMs, albeit in the form of the observed low-level cell clones, may increase the risk of acquiring additional mutations and represents the first step in potentially evolving leukemia. Individuals with CHIP have an 11–13 times increased risk of developing hematologic neoplasia, compared with the general population. This is because the acquisition of subsequent, disease driving mutations occurs at a much higher rate in people already affected by clonal expansion, initiated by early mutations. All-cause mortality was also observed with higher incidence among individuals with detectable CH, possibly due to higher risk of death from cardiovascular disease [2, 3]. A recent study demonstrated almost the doubling of the risk of coronary heart disease in humans in case of CHIP in peripheral-blood cells [19]. However, the absolute risk of developing a malignant hematologic disease is low, with the rate of progression being 0.5–1% per year [4]. Further research should focus on the underlying mechanisms which transform clonal haematopoiesis from a seemingly common and benign to a relatively rare malignant state.

In the cohort of healthy elderly individuals *DNMT3A* mutations were the most frequent (14%; 7/50), with one person harboring two different *DNMT3A* mutations in independent clones. This is in accordance with previous studies, which also identified *DNMT3A* as one of the most frequently mutated epigenetic regulator genes [1–3]. Using a custom-designed highly sensitive *DNMT3A* NGS assay age-dependence of *DNMT3A* mutations was assessed. In the whole cohort of young and elderly healthy persons (*n* = 210), 10 out of 13 *DNMT3A* mutations were detected in individuals older than 60 years, displaying an age-dependent increase of *DNMT3A* mutation prevalence. *DNMT3A* mutations are suggested to arise early in AML, leading to a clonally expanded pool of pre-leukemic HSCs [20]. Persistence of low-level *DNMT3A* mutations during remission in AML patients further supports the existence of pre-leukemic stem cells with CHDMs, which then represent a reservoir of pre-leukemic clones that can provoke a relapse [21].

Previous studies have shown that *DNMT3A* is among the most frequently mutated genes in AML, MPN, MDS, and adult-early T-cell precursor acute lymphoblastic leukemia (ETP-ALL) [16, 22–26]. Approximately 22% of cytogenetically normal acute myeloid leukemia (CN-AML) patients carry a *DNMT3A* mutation, from which about 60% are localized in the catalytic domain, at the R882 hotspot [22, 27]. These mutations exhibit dominantly negative functional effects, decreasing enzymatic activity, thus causing hypomethylation of target genes [28–30]. Their presence is correlated with an adverse outcome and a higher therapy-resistance rate in AML [22, 27]. Such hotspot mutations were also detected within the cohort of healthy individuals in this study, suggesting that they are an early event occurring in the pathogenesis of different hematological diseases.

Recent data further suggest a temporal hierarchy in *DNMT3A* and *TET2* mutations’ occurrence. Whereas *DNMT3A* mutations origin in multipotent stem cells, *TET2* mutations occur mainly in myeloid cells [31]. Somatic mutations of *DNMT3A* and *TET2* were also associated with accelerated atherosclerosis, thus increasing the risk of cardiovascular disease mediated by inflammatory mechanisms [32, 33]. This finding indicates that CHDMs may predispose to both hematologic and non-hematologic diseases and might represent a valuable predictive and prognostic factor.

Furthermore, gene expression analysis showed significant age-dependent decrease of *DNMT3A* expression. This decrease is independent of the mutation status, implying that other factors apart from genomic alterations, such as changes in the bone marrow niche and an aging cellular background may play an important role in the *DNMT3A* expression rate and thus function. This is in correspondence with the higher prevalence of CH and hematologic malignancies in elderly [1–3]. Decreased *DNMT3A* expression rate with age may contribute to the bias of HSCs toward self-renewal, by affecting expression of downstream targets as shown by Challen et al. who found an upregulation of “HSC fingerprint” genes in *DNMT3A*-null HSCs [5]. These genes, such as *NR4A2*, *PDK4*, *VASN*, and *PRDM16* are normally expressed in HSCs but not in differentiated blood cells [34], suggesting that *DNMT3A* is required to suppress the stem cell program in HSCs to permit differentiation. Furthermore, multipotency genes including *RUNX1*, *GATA3*, *PBX1*, and *CDKN1A* were also highly expressed in *DNMT3A*-null HSCs, opposite to decreased expression of essential differentiation factors, such as those encoded by *FLK2*, *IKAROS*, *SFPI1*, and *MEF2C* [5]. Taken together, altered expression of downstream target genes due to lower *DNMT3A* expression, resulting from mutations or other age-related factors, may promote CH.

To further investigate the potential clonal selection of cells harboring *DNMT3A* mutations a mouse model was established. For that purpose, xenotransplantations of bone marrow cells from two healthy elderly patients with *DNMT3A* mutations were performed and the mutations’ dynamics assessed over a period of 8 months. *DNMT3A* mutations displayed relatively stable kinetics. Mice xenografts from individual A, with an initial VAF of 23.1%, displayed a median VAF of 21.8% (range: 10.8–30.8%), showing stable kinetics. The initial VAF of 6.3% of individual B also remained stable in one out of three transplanted mice (median VAF 6.2%). For the remaining two mice the mutational burden may have been below the detectability level of the pyrosequencing assay (5%). Comparing this data with the longitudinal study on healthy individuals we can conclude that CHDMs, both in humans and a mouse model of aging hematopoiesis, are displaying relatively stable mutations kinetics, without clonal selection of the cells carrying mutations or progressing to overt hematological disease. This again suggests, that the presence of CHDMs itself is insufficient to initiate clonal selection and expansion without an additional influence of other intrinsic and/or extrinsic factors.

In conclusion, these findings indicate that the appearance of low-level clones with mutations in leukemia-associated genes is a common age-associated phenomenon. Occurrence of these mutations within epigenetic regulator genes and the RNA-splicing machinery may represent a premalignant condition in the development of hematologic cancers and predispose to other aging-associated disorders. *DNMT3A* mutations were the most common mutations identified in elderly individuals but were also apparent in younger healthy people, albeit less frequently. Further studies should investigate which compensatory mechanisms inhibit selection of cells carrying mutations, promoting a stage of clonal size stability in healthy elderly individuals in contrast to patients with overt myeloid disorders. Interestingly, this study shows an age-related decrease in *DNMT3A* expression and highlights that in addition to CHDMs, aberrant expression may be a feature of the aging hematopoietic system. Further studies should investigate whether other leukemia-associated genes show similar patterns.

### Supplementary information

Supplemental Material
