# Supplementary material for: Prevalence and dynamics of clonal hematopoiesis caused by leukemia-associated mutations in elderly individuals without hematologic disorders
Source: Leukemia. 2020 May 26;34(8):2198–205. doi: 10.1038/s41375-020-0869-y (PMC7387320; doi:10.1038/s41375-020-0869-y)
Supplement: Supplementary file 1 — Supplemental Material [file 41375_2020_869_MOESM1_ESM.docx]

**Supplemental Data**

**Table SI** Primers for qPCR

| **Primer** | **Sequence (5’→3’)** | **Amplicon length** |
| --- | --- | --- |
| *DNMT3A*_exon 17-18_forward | CTCGGAGGTGTGTGAGGACT | 357 bp |
| *DNMT3A*_exon 17-20_reverse | TCAATCATCACAGGGTTGGA |  |
| *GUSB*_forward | AGAAACGATTGCAGGGTTTCAC | 205 bp |
| *GUSB*_reverse | CCGAGTGAAGATCCCCTTTTTA |  |

**Table SII** Primers for Pyrosequencing

| **Primer** | **Sequence** | **Amplicon length** |
| --- | --- | --- |
| *DNMT3A*_S837X_F | Bio-GCAAAGTGAGGACCATTACTACGA | 122 bp |
| *DNMT3A*_S837X_R | CGGGTACCTTTCCATTTCAGT |  |
| *DNMT3A*_S837X_S | CCCTGCTTTATGGAGTT |  |
| *DNMT3A*_D768H_F | CGGCCCAAGGAGGGAGAT | 238 bp |
| *DNMT3A*_D768H_R | Bio-CCTGGGGCTTCCCAAACA |  |
| *DNMT3A*_D768H_S | GGCGTTAGTGACAAGAG |  |

**Table SIII** *DNMT3A* mutations in bone marrow of healthy individuals

| **Gene** | **Nucleobase**  **change** | **Amino acid change** | **Info** |
| --- | --- | --- | --- |
| *DNMT3A* | c.2510C>A | p.Ser837Ter | SNP (rs1374267987) Consequence: stop gained |
| *DNMT3A* | c.2302G>C | p.Asp768His | SNP (rs767983115) Sift: deleterious (0) PolyPhen: probably damaging (1)  Consequence: missense variant |
